# A comparison of IROC and ACDS on‐site audits of reference and non‐reference dosimetry

**DOI:** 10.1002/mp.13800

**Published:** 2019-10-25

**Authors:** Jessica Lye, Stephen Kry, Maddison Shaw, Francis Gibbons, Stephanie Keehan, Joerg Lehmann, Tomas Kron, David Followill, Ivan Williams

**Affiliations:** ^1^ Australian Clinical Dosimetry Service ARPANSA Melbourne Australia; ^2^ Imaging and Radiation Oncology Core Houston QA Center MD Anderson Cancer Center Houston TX USA; ^3^ Sunshine Coast Hospital and Health Service Birtinya Qld Australia; ^4^ Department of Radiation Oncology Calvary Mater Newcastle Newcastle Australia; ^5^ Peter MacCallum Cancer Centre Melbourne Australia

**Keywords:** Australian Clinical Dosimetry Service, dosimetry audit, Imaging and Radiation Oncology Core, international comparison, quality assurance, Radiation Therapy audit

## Abstract

**Purpose:**

Consistency between different international quality assurance groups is important in the progress toward similar standards and expectations in radiotherapy dosimetry around the world, and in the context of consistent clinical trial data from international trial participants. This study compares the dosimetry audit methodology and results of two international quality assurance groups performing a side‐by‐side comparison at the same radiotherapy department, and interrogates the ability of the audits to detect deliberately introduced errors.

**Methods:**

A comparison of the core dosimetry components of reference and non‐reference audits was conducted by the Imaging and Radiation Oncology Core (IROC, Houston, USA) and the Australian Clinical Dosimetry Service (ACDS, Melbourne, Australia). A set of measurements were conducted over 2 days at an Australian radiation therapy facility in Melbourne. Each group evaluated the reference dosimetry, output factors, small field output factors, percentage depth dose (PDD), wedge, and off‐axis factors according to their standard protocols. IROC additionally investigated the Electron PDD and the ACDS investigated the effect of heterogeneities. In order to evaluate and compare the performance of these audits under suboptimal conditions, artificial errors in percentage depth dose (PDD), EDW, and small field output factors were introduced into the 6 MV beam model to simulate potential commissioning/modeling errors and both audits were tested for their sensitivity in detecting these errors.

**Results:**

With the plans from the clinical beam model, almost all results were within tolerance and at an optimal pass level. Good consistency was found between the two audits as almost all findings were consistent between them. Only two results were different between the results of IROC and the ACDS. The measurements of reference FFF photons showed a discrepancy of 0.7% between ACDS and IROC due to the inclusion of a 0.5% nonuniformity correction by the ACDS. The second difference between IROC and the ACDS was seen with the lung phantom. The asymmetric field behind lung measured by the ACDS was slightly (0.3%) above the ACDS's pass (optimal) level of 3.3%. IROC did not detect this issue because their measurements were all assessed in a homogeneous phantom. When errors were deliberately introduced neither audit was sensitive enough to pick up a 2% change to the small field output factors. The introduced PDD change was flagged by both audits. Similarly, the introduced error of using 25° wedge instead of 30° wedge was detectible in both audits as out of tolerance.

**Conclusions:**

Despite different equipment, approach, and scope of measurements in on‐site audits, there were clear similarities between the results from the two groups. This finding is encouraging in the context of a global harmonized approach to radiotherapy quality assurance and dosimetry audit.

## 1 Introduction

Independent audits of dosimetry in radiotherapy clinics are an excellent quality improvement tool for detecting systemic errors in dosimetry and encouraging consistency in radiotherapy practice. Dosimetry audits are recognized as international best practice for departmental quality assurance and clinical trial accreditation and have uncovered systemic problems with radiotherapy dose determination, such as dosimetric inaccuracies in heterogeneous dose calculations^1^, ^2^, ^3^, ^4^ and small field dose calculations,^5^, ^6^, ^7^ as well as identifying unique errors in calibration and dose determination at individual clinics.^8^, ^9^

Dosimetry audits can test different elements of the radiotherapy chain, including a simple check of reference dosimetry (Level I audit), increasing in complexity to an end‐to‐end dose delivery evaluation (Level III).^10^, ^11^, ^12^ An intermediate (Level II) audit is one that probes the commissioning data and development of the treatment planning system (TPS) beam model.^13^ Such a clinical dosimetry audit can take many forms, as any type of dosimetric measurement that falls between reference dosimetry and full end‐to‐end scanning, planning, and delivery style audits could be classified as a Level II audit. Ideally, for a given level of audit, the audit should be sensitive to the detection of dosimetric deficiencies regardless of the means by which it is conducted. This means, implicitly, that audits performed by different groups worldwide should, despite differences in methodology, be consistent in their findings. Indeed, consistency between different international QA groups is a key focus and concern of such groups as the Global Harmonisation Group (GHG; http://www.rtqaharmonization.com).^14^

The ability of different audit methodologies to produce comparable results is not given in assessing radiotherapy performance. Indeed, many recent studies have shown inconsistent (and inaccurate) performance of common quality assurance (QA) tools, particularly in the assessment of IMRT.^15^, ^16^, ^17^, ^18^, ^19^ Therefore, a comparison of the core components of on‐site Level I and II audits was conducted by the Imaging and Radiation Oncology Core Houston QA Center (IROC, Houston, USA) and the Australian Clinical Dosimetry Service (ACDS, Melbourne, Australia). This study evaluated if the different dosimetric approach from two groups on opposite sides of the world, and using separate primary dosimetry standards and calibration protocols,^20^, ^21^ could comparably assess radiotherapy quality and identify errors deliberately introduced in the planning system. This was done through a head‐to‐head set of measurements conducted at an Australian radiation therapy facility in Melbourne.

## 2 Materials and Methods

### 2.1 Audit components

The dosimetric components of IROC and ACDS on‐site audits of photon beams include an evaluation of: beam output, output factors (including for small fields), depth dose data (for different field sizes), off‐axis factors, and wedge factors. Electron output is also evaluated. The specific components for each group are shown in Table 1. From this table, it is clear that many similar components of clinical dosimetry are tested, but that these are done with different implementations, equipment, and methodologies.

**Table 1:** List of audit cases for non‐reference dosimetry.

Tested dosimetric component of beam model	IROC case			ACDS case		
Output factors	6 × 6 15 × 15 20 × 20 30 × 30			3 × 3 20 × 20		
Small field output factors	6 × 6 4 × 4 3 × 3 2 × 2			3 × 3 2 × 2 1 × 1		
PDD	6 × 6 5 cm 10 cm 15 cm 20 cm	10 × 10 d_max_ 5 cm 10 cm 15 cm 20 cm	20 × 20 5 cm 10 cm 15 cm 20 cm	12 × 12 8 cm 15 cm	12 × 12 EDW 8 cm 15 cm	12 × 12 Lung 8 cm 15 cm
Wedge (physical and enhanced dynamic)	60° EDW 10 × 10 45° EDW 10 × 10 45° EDW 15 × 15 60° Physical 10 × 10 45° Physical 10 × 10			60° EDW 10 × 6 30° EDW 12 × 12		
Off‐axis factors	5 cm left 10 cm left/right 10 cm toward/away 15 cm left			All fields (2D array measurements)		
Asymmetric fields	*N/A*			Asymmetric 10 × 6		
Lung	*N/A*			12 × 12 30° EDW 12 × 12 60° EDW 10 × 6		
Electrons PDD	50% depth dose 80% depth dose			*N/A*		

2D, two‐dimensional; ACDS, Australian clinical dosimetry service; IROC, imaging and radiation oncology core.

Both the IROC and the ACDS include additional components in their on‐site audit. For example, IROC performs a picket fence test of the MLC, mechanical checks on the linac, and a Winston–Lutz test for IGRT. The ACDS performs test cases from TG‐119^22^ and MLC commissioning tests including the “Chair” test^23^ and the FOURL.^25^ While these tests are important components of an independent audit, this work focuses on core conformal dosimetry components and analysis listed in Table 1.

Dosimetric audits cover multiple modalities of radiation therapy dose delivery and the approach to covering modalities differs between auditing groups. The IROC on‐site audit covers a number of modalities in a single visit. The ACDS Level Ib audits cover reference beams and the ACDS Level II audit covers non‐reference conformal photons, IMRT, and VMAT. This work compares the ACDS Level Ib and Level II audits to match the modalities covered by the IROC on‐site audit.

### 2.2 Audit materials and methods

**Figure 1:**
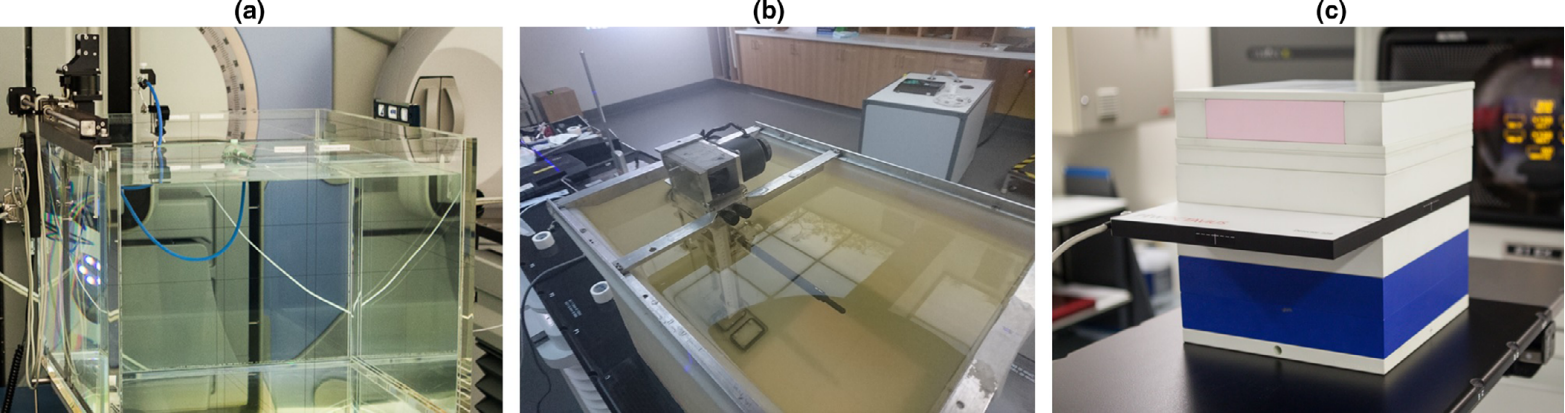
The water tank and ion chambers (a) used by the Australian clinical dosimetry service (ACDS) for reference dosimetry, the ion chamber in a scanning water tank (b) used by imaging and radiation oncology core for both reference and non‐reference dosimetry, and the array of ion chambers used by the ACDS in a slab phantom (c) of solid water and lung‐equivalent plastic for non‐reference dosimetry are shown.

The IROC determines the absorbed dose to water per monitor unit, for megavoltage photon and electron beams, under the Radiation Oncology facility's reference conditions following the AAPM TG‐51 protocol. IROC brings all their own equipment on‐site including their own custom one‐dimensional scanning water tank [Fig. 1(a)]. Farmer type ionization chambers (primarily the Exradin A12, Standard Imaging Middleton, WI) are used for reference photon, electron, and FFF dose measurements. Charge is measured with a Standard Imaging model MAX 4000 electrometer. All ion chambers and electrometers are calibrated every second year and checked before each site visit by an Accredited Dosimetry Calibration Laboratory.

Non‐reference dose assessments are conducted by IROC based on point dose measurements in the same water phantom [Fig. 1(b)]. The institution, using their TPS, calculates the dose in a virtual water phantom to the points and conditions prescribed by IROC (Table 1). Measurements are then conducted primarily with the same reference A12 Farmer‐type ion chamber. However, for the measurement of small field output factors, the Exradin Micropoint A16 ion chamber (Standard Imaging Middleton, WI) is used as the detector.

The ACDS determines absorbed dose to water per monitor unit, for megavoltage photon and electron beams, under the Radiation Oncology facility's reference conditions following the IAEA TRS‐398 Code of Practice. Measurements are conducted in the facility's water tank [Fig. 1(a)]. All other equipment is brought on‐site by the ACDS. Farmer type ionization chambers such as the PTW model TW30013 (PTW Freiburg, Germany) are used for reference photon and FFF dose measurements, and the PTW model 34001 Roos ionization chambers is used for reference electron dose measurements. A nonuniformity correction is applied for FFF measurements to account for the change in profile over the length of a farmer chamber (0.3% and 0.5% for 6FFF and 10FFF, respectively.^26^ Charge is measured with a PTW UNIDOS webline reference class electrometer. The ACDS uses ion chamber calibration factors traceable to the Australian Primary Standard of absorbed dose and determined in high‐energy beams of similar quality (referred to as “Directly measured”), as recommended by the TRS‐398 code. The Farmer type chambers are secondary standards, directly calibrated in comparison with the Australian primary standard. The Roos chamber and the electrometers are also calibrated by the Australian Radiation Protection and Nuclear Safety Agency (ARPANSA).

Non‐reference dose assessments were conducted by the ACDS over selected points and planes within a “slab” geometry phantom [Fig. 1(c)]. A CT of the slab phantom is supplied to the Facility for treatment planning based on the multiple cases prescribed by the ACDS (Table 1). Dosimetry measurements are made in a custom phantom of CIRS solid water (CIRS Inc, Norfolk, VA, USA), using a PTW Octavius 1500 two‐dimensional (2D) ionization chamber array (PTW Freiburg, Germany) as a primary detector and supporting measurements with IBA model CC13 ionization chambers (IBA Dosimetry GmbH, Schwarzenbruck, Germany). The 2D array is calibrated against a Farmer type ionization chamber, which is traceable to the Australian primary standard of absorbed dose. The PTW 60019 microDiamond was used as the primary detector during the measurement of the small field output factors, although at the time of print, the ACDS small field modality was in field trial status and no acceptance or out of tolerance outcome was defined.

The uncertainty in the absolute and relative dose measurements is similar for all of the ion chamber‐based large field measurements in this study. The relative standard uncertainty in the dosimetry measurements is estimated to be 1.5% from the TRS‐398 calculated uncertainty for high‐energy photon beams.

### 2.3 Audit comparison

The two groups performed their on‐site audits, each following their own standard procedures, on the same day in a clinical radiotherapy facility in Melbourne, Australia. The measurements were conducted on a Varian TrueBeam Linear Accelerator (Varian Medical Systems, Palo Alto, CA, USA), commissioned for use with the Eclipse Treatment Planning System v13.6.30 (Varian Medical Systems, Palo Alto, CA, USA). The 6 MV photon beam was fully audited, while (due to time limitations) the 10 FFF and 18 MV beams were only evaluated in terms of beam calibration. Electron beams with energies of 6, 9, 12, 15, and 18 MeV were also evaluated.

In order to evaluate and compare the performance of these audits when radiotherapy deficiencies are present, known errors in PDD, EDW, and small field output factors were introduced into the 6 MV beam model to simulate potential commissioning/modeling errors. To simulate an error in 6 MV beam model PDD, the TPR_20/10_ ratios were increased by 3%. To simulate an error in the EDW commissioning, the plans with a 30° EDW were replaced with a 25° EDW. Finally, the output factors were increased for the 3 × 3, 2 × 2, and 1 × 1 cm^2^ field sizes by 2.4%, 2.1%, and 2.0%, respectively. The slight differences in size of the error were due to rounding differences. The audit plans were recalculated with the erroneous beam models and compared to the original measurements. Both audits were tested for their sensitivity in detecting these errors. The errors were chosen so as to cover a range of beam model components, and also to cover a range of error magnitude. The output factors were increased by the smallest amount, of approximately 2%. As discussed below, 2% is the smallest tolerance used by the two audit groups in assessing the beam modeling. The PDD was adjusted by a slightly larger amount of 3%, a tolerance used by both groups in assessing beam modeling. Finally, an obvious beam model error of using the wrong wedge profile in commissioning was investigated. The audit teams did not know what the introduced errors were prior to performing the audits.

### 2.4 Audit tolerances and uncertainties

The measurement uncertainties in this report are evaluated using the addendum to TG‐51.^24^ The reference dosimetry measurements for both groups are performed with a reference class farmer chamber with an expanded uncertainty of 1.8% (2σ). Also measured are relative quantities such as output factors, wedge factors, and PDD factors. Taking into account the correlation between uncertainty components relating to the chamber calibration factor and some correction factors, the uncertainty in relative dose measurement using a farmer chamber is estimated to be 0.8% (2σ). The ACDS measures the relative doses with an ionization chamber array. Increased uncertainty is included in the charge history, chamber stability, and irradiation history for assessment of uncertainty in the array‐based relative dose measurement, resulting in expanded uncertainty of 1.6% (2σ). Note that all of these uncertainties are assessing uncertainty in the audit group measurement only and does not include uncertainty in facility measurement or TPS calculation.

The IROC and the ACDS have different criteria for defining if an audit result is acceptable, or within tolerance. For reference dose measurements, IROC employs a ±3% tolerance criterion, that is, the ratio of IROC measured to institution stated dose output must be within 3%. All other dose measurements are evaluated as relative factors, that is, output factors, wedge factors, and PDD factors. Given the correlated uncertainties in the measurement and the reduction in uncertainty budget for relative measurements, IROC employs a ±2% tolerance criterion on the ratio of IROC measured to institution calculated relative factors. Results outside these tolerances are presented to the institution as actionable deficiencies with associated recommendations. For each field size measured by the IROC on a particular machine, the product of the IROC/institution ratios for the relative dose factors are also evaluating by selecting first the minimum ratios and then the maximum ratios which could potentially be combined in calculating tumor dose. The products of these ratios give the range of potential disagreement between IROC and institution on the calculation of stated tumor dose for cooperative trials and should be within ±5.0%.

The ACDS employs an out of tolerance criteria of ±2.1% for reference dose measurements of the reference beam calibration. For reference dosimetry comparisons, there is no treatment planning and ACDS measured dose is compared to the facility measured dose. For non‐reference measurements, the ACDS compares the TPS predicted dose to absolute dose measurements. For direct comparison to the TPS predicted dose, the ACDS employs an out of tolerance criterion of ±5.0%. The ACDS simply looks at all points measured in the field, and scores on whether the dose, uncorrected for output of the day, is within the tolerance limit. The philosophy is to measure the dose that would be delivered to a patient. A drawback of this approach is that multiple errors occurring in positive and negative directions may cancel out and be missed. However, the range of fields investigated and listed in Table 1 is designed to drill down into individual effects similar to those explicitly investigated by IROC. ACDS also has two levels of Pass; Pass (optimal level) and Pass (action level), allowing for identification of results that can be improved, even if the beam is overall acceptable. Optimal level occurs for dosimetry within or equal to ±1.4% and ±3.3% for reference and non‐reference dosimetry, respectively.

Throughout this report, any result that is not optimal and would result in a recommendation (being outside IROC's tolerance or the action level as categorized by the ACDS) will be noted by “R.”

## 3 Results and Discussion

### 3.1 Reference dosimetry

The results for reference dosimetry measurements are shown in Table 2, and demonstrate excellent agreement between the two audits, particularly for photon beams. The measurements of FFF photons show a slight discrepancy due to the inclusion of a 0.5% nonuniformity correction included by the ACDS^26^ but not included by IROC at the time of this audit. The nonuniformity correction was not included by the facility, which led to an action level result in the ACDS audit. Measurements of reference dosimetry in electron beams between the two groups also showed good agreement, with a maximum discrepancy of 0.8%. The results of the reference modalities confirm the success of the international system of dosimetry^27^: good agreement in measurements from the two groups was seen despite following different dosimetry protocols; IROC uses the AAPM TG‐51 protocol while the ACDS uses IAEA TRS‐398^28^ and traceability to different primary standards.

**Table 2:** Audit results for the 10 × 10 cm^2^ reference beam outputs, showing the ratio of audit measured output to that determined by the facility.

Energy	IROC/facility	ACDS/facility	Difference IROC‐ACDS
Photons			
6	1.011	1.010	0.1%
10FFF	1.009	1.016^R^	−0.7%
18	1.012	1.012	0.0%
Electrons			
6	0.991	0.998	−0.7%
9	0.989	0.995	−0.6%
12	0.990	0.995	−0.5%
15	0.988	0.993	−0.5%
18	1.002	0.994	0.8%

ACDS, Australian clinical dosimetry service; IROC, imaging and radiation oncology core.

R Nonoptimal result resulting in recommendation.

### 3.2 Output factors

The ratio of measured output factors to the TPS calculated output factors at the facility for both audit groups is shown in Table 3. Good agreement was seen between IROC and the ACDS for the 20 × 20 cm^2^ and 3 × 3 cm^2^ reference cases. For the small field (2 × 2 cm), a difference of 0.8% was seen, which is reasonable considering the complexities of small field dosimetry measurements.

**Table 3:** Ratios of measured (by IROC or the ACDS) to calculated (by the facility) output factors for the 6 MV beam.

Size	IROC/facility	ACDS/facility	Difference IROC‐ACDS
Small field chambers			
1 × 1		1.032^a^	
2 × 2	0.999	1.007^a^	−0.8%
3 × 3	0.999	1.001^a^	−0.2%
4 × 4	0.998		
6 × 6	1.008		
Farmer and array			
6 × 6	1.013		
15 × 15	1.012		
20 × 20	1.011	1.005	0.6%
30 × 30	1.004		

ACDS, Australian clinical dosimetry service; IROC, imaging and radiation oncology core.

a Field‐trial status only. No tolerances have been assigned to the ACDS small field modality.

The results from both audits after the introduction of deliberate errors in the form of increasing the small field output factors in the 6 MV beam model are shown in Table 4. While the results of both audits did pick up the differences, the adjustments made to the beam model were not large enough to show as deficiencies in either group's audit, and in the case of the 1 × 1 for ACDS measurements, the plan adjustment improved the agreement due to the direction of the original variation between plan and measurement.

**Table 4:** Output factors with error introduced adjusted plans for the 6 MV beam.

Size	IROC/facility plan original	IROC/facility error plan	ACDS/facility plan original	ACDS/facility error plan
1 × 1			1.032^a^	1.001^a^
2 × 2	0.999	0.976	1.007^a^	0.988^a^
3 × 3	0.999	0.978	1.001^a^	0.981^a^
4 × 4	0.998	0.992		

ACDS, Australian clinical dosimetry service; IROC, imaging and radiation oncology core.

a Field‐trial status only. No tolerances have been assigned to the ACDS small field modality.

### 3.3 Percent depth dose

IROC performs a thorough set of point dose measurements at different depths and fields sizes to assess the quality of the facility's modeling of PDD. All measured PDD values are normalized to the institution's value at 10 cm depth (for that field size).

**Figure 2:**
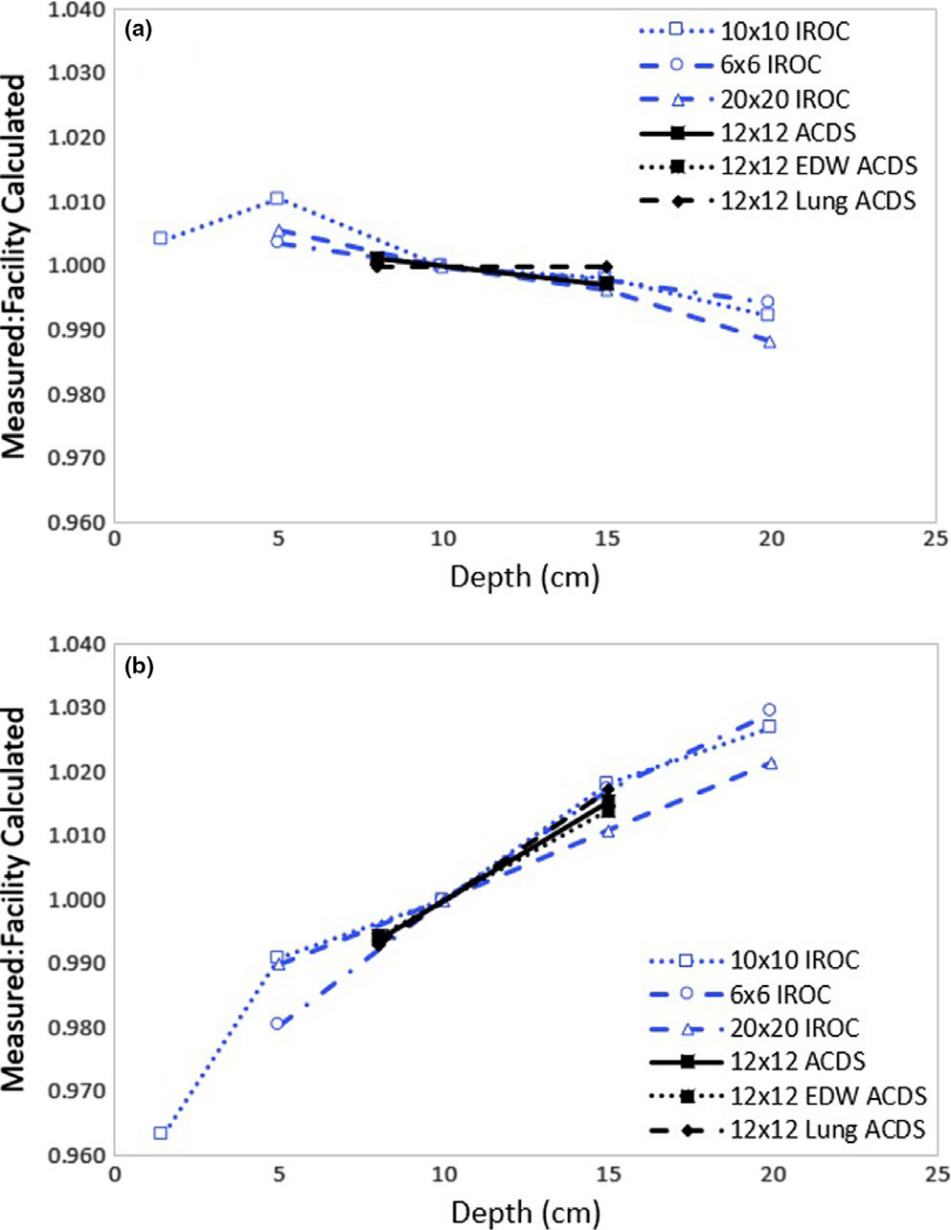
The ratio of measured to facility planned PDD factors with the actual plans are shown in 2a, and panel 2b shows the PDD comparison results for the plan with the introduced error.

In contrast, the ACDS measures the dose at two depths (8 and 15 cm) for select cases: with and without the presence of heterogeneity and wedges for a 12 × 12 cm field size. For the purposes of comparing with the IROC data, the ACDS‐generated PDDs were normalized to a depth of 10 cm. Figure 2(a) shows the ratio of measured to TPS PDD factors for both IROC and ACDS. There is excellent agreement between the two audits assessment of PDD, with good plan modeling of PDD observed in both audits.

The impact of an introduced error in the 6 MV beam model (based on increasing the TPR_20/10_ ratios by 3%) is shown in figure Fig. 2(b). The error is seen in both audits as the measured/calculated ratio deviates substantially from unity.

The outcomes of the PDD audit relative to tolerances when compared to the original/clinical plan and the plan containing the introduced error are shown in Table 5. For the original plan, both the IROC and ACDS audits performed with a homogeneous water phantom found all results to be optimal. Both audits flagged the introduced PDD error as being deficient.

**Table 5:** Audit outcomes for testing PDD component of beam model for 6 MV only. The ACDS results include both the central axis dose variations and the maximum dose difference across the two‐dimensional (2D) plane in field.

IROC/Facility PDD factors central axis			ACDS/facility dose central axis (max dose variation across 2D plane)		
Size, depth	Original plan	Error plan	Case	Original plan	Error plan
*PDD in water only*					
6 × 6, 5 cm	1.004	0.980	12 × 12, 15 cm	1.009 (1.012)	1.030 (1.032)
6 × 6, 10 cm	1.000	1.000	12 × 12, 8 cm	1.013 (1.015)	1.008 (1.010)
6 × 6, 15 cm	0.998	1.017	12 × 12 EDW, 15 cm	1.008 (1.011)	1.027 (1.030)
6 × 6, 20 cm	0.994	1.029^R^	12 × 12 EDW, 8 cm	1.012 (1.015)	1.007 (1.009)
10 × 10, dmax	1.004	0.963^R^	10 × 6, 15 cm	1.014 (1.015)	1.032 (1.034)^R^
10 × 10, 5 cm	1.010	0.991	10 × 6 EDW, 15 cm	1.012 (1.012)	1.030 (1.030)
10 × 10, 10 cm	1.000	1.000	3 × 3, 15 cm	1.002 (1.002)	1.023 (1.023)
10 × 10, 15 cm	0.998	1.018			
10 × 10, 20 cm	0.992	1.027^R^			
20 × 20, 5 cm	1.006	0.990			
20 × 20, 10 cm	1.000	1.000			
20 × 20, 15 cm	0.996	1.011			
20 × 20, 20 cm	0.988	1.022			
*PDD with lung*					
			12 × 12 lung, 15cm	1.008 (1.011)	1.012 (1.015)
			12 × 12 lung, 8 cm	1.008 (0.988)	0.988 (0.971)
			12 × 12 EDW lung, 15 cm	1.008 (1.012)	1.012 (1.015)
			12 × 12 EDW lung, 8 cm	1.007 (0.988)	0.986 (0.970)
			10 × 6 lung, 15 cm	1.014 (1.036)^R^	1.017 (1.037)^R^
			10 × 6 EDW lung, 15 cm	1.012 (1.035)^R^	1.014 (1.036)^R^

ACDS, Australian clinical dosimetry service; IROC, imaging and radiation oncology core.

R Nonoptimal result resulting in recommendation.

For the ACDS audits with lung present, the results with the original clinical plan were mainly optimal, but with nonoptimal results observed in the asymmetric fields 10 × 6 cm^2^ with and without wedge. The individual nonoptimal points were near the water and lung juncture. An interesting result from the ACDS audit is the observed change when the lung slab is present and the PDD error is introduced. When the PDD error was introduced into the model, the lung case (no wedge) measured at 8 cm depth was sensitive to the change, with central axis dose difference increasing by 2%. The measurement at 15 cm depth was insensitive to the introduced PDD error. In contrast, with the homogeneous phantom, the central axis dose difference at 15 cm depth decreases by 2% and the 8 cm depth was insensitive.

The ACDS audit does not correct for the measured daily output. If the ACDS were to correct for the measured daily output of 1.012 cGy/MU for 6X, the results shown in Table 5 would improve. The results from the plan with introduced errors would then all be in the optimal range.

In comparing the two audit results, strengths and weaknesses in each are seen. A relative strength of the IROC audit is that the range of discrepancy in the PDD values (for the introduced error) identified by IROC was larger due to their more extensive testing of different depths and field sizes. Measurements at shallower and deeper depths identify more problems than those close to the normalization depth. A relative strength of the ACDS audit is the inclusion of the lung case, which showed a systematic behavior with the introduced PDD error.

The IROC additionally measured electron depths of 80% and 50% doses. All measurements agreed to within 1 mm to the institution's stated depth. The ACDS does not measure electron depth dose data.

### 3.4 Wedge

The IROC and ACDS audits both assess accuracy of the dose calculation in the presence of wedges; IROC calculates wedge factors, and ACDS measures the dose delivered in a wedged field directly without normalization to the open field. In the comparison, IROC measured the 45° and 60° wedges with both a physical wedge and enhanced dynamic wedges (EDW). ACDS measured the EDW for 30° and 60° wedges. The results from the IROC and ACDS wedge measurements in a homogeneous water phantom are shown in Table 6. The ACDS results are presented as the ratio between measured and TPS calculated wedge factors (for direct comparison with IROC results) and as the ratio of measured and TPS calculated doses. Both audits saw highly consistent results in the ratio of measured vs planned wedge factors. Both the IROC and the ACDS found optimal results for the homogeneous water phantom based on the original (clinical) plan, indicating good modeling of the wedges.

**Table 6:** Audit outcomes for testing the wedge component of beam model for a 6 MV beam in a homogeneous water phantom. IROC reports the ratio of measured to plan wedge factors. ACDS measurements have also been reported as ratio of wedge factors. The ACDS result in the final column includes both the central axis (CAX) dose variations and the maximum dose difference across the two‐dimensional (2D) plane in field.

IROC case	IROC/facility WF central axis	ACDS case	ACDS/facility wedge factor central axis	ACDS/facility dose CAX (Max)
EDW				
60° EDW 10 × 10, 10 cm	1.002	60° EDW 10 × 6, 15 cm	1.002	1.012 (1.012)
45° EDW 10 × 10, 10 cm	1.000	30° EDW 12 × 12,15 cm	1.001	1.008 (1.011)
45° EDW 15 × 15, 15 cm	1.000	30° EDW 12 × 12, 8 cm	1.001	1.012 (1.015)
Physical wedge				
60° upper	0.998			
45° upper	1.006			

ACDS, Australian clinical dosimetry service; IROC, imaging and radiation oncology core.

To evaluate the sensitivity of the two audits with respect to identifying wedge issues, the plans with a 30° EDW were replaced with a 25° EDW. IROC did not directly measure the 30° EDW due to time limitations during the comparison. The wedge factors measured by IROC showed minimal variation from the facility plan (0.0%–0.2%), and the ACDS measurements including the 30° also showed minimal variation (0.1%). Therefore, it was assumed the IROC measurement of the 30° EDW would also show minimal variation (0.0%) from the actual plan for the purpose of this sensitivity test. Table 7 shows the outcomes form the audits when the plan with the incorrect wedge was introduced. The incorrect wedge was flagged in both audits.

**Table 7:** Audit outcomes for testing the wedge component of beam model for the 6 MV beam in a homogeneous water phantom with the actual plans and the adjusted plans with incorrect wedge profile included.

IROC/facility WF central axis			ACDS/facility dose central axis (max dose variation across 2D plane)		
Size, depth	Plan actual	Error plan	Case	Plan actual	Error plan
30° EDW 10 × 10, 10 cm	1.000^a^	0.972^R^	30° EDW 12 × 12, 15 cm	1.008 (1.011)	0.967 (0.939)^R^
			30° EDW 12 × 12, 8 cm	1.012 (1.015)	0.971 (0.940)^R^

ACDS, Australian clinical dosimetry service; IROC, imaging and radiation oncology core.

a Not measured.

R Nonoptimal result resulting in recommendation.

**Figure 3:**
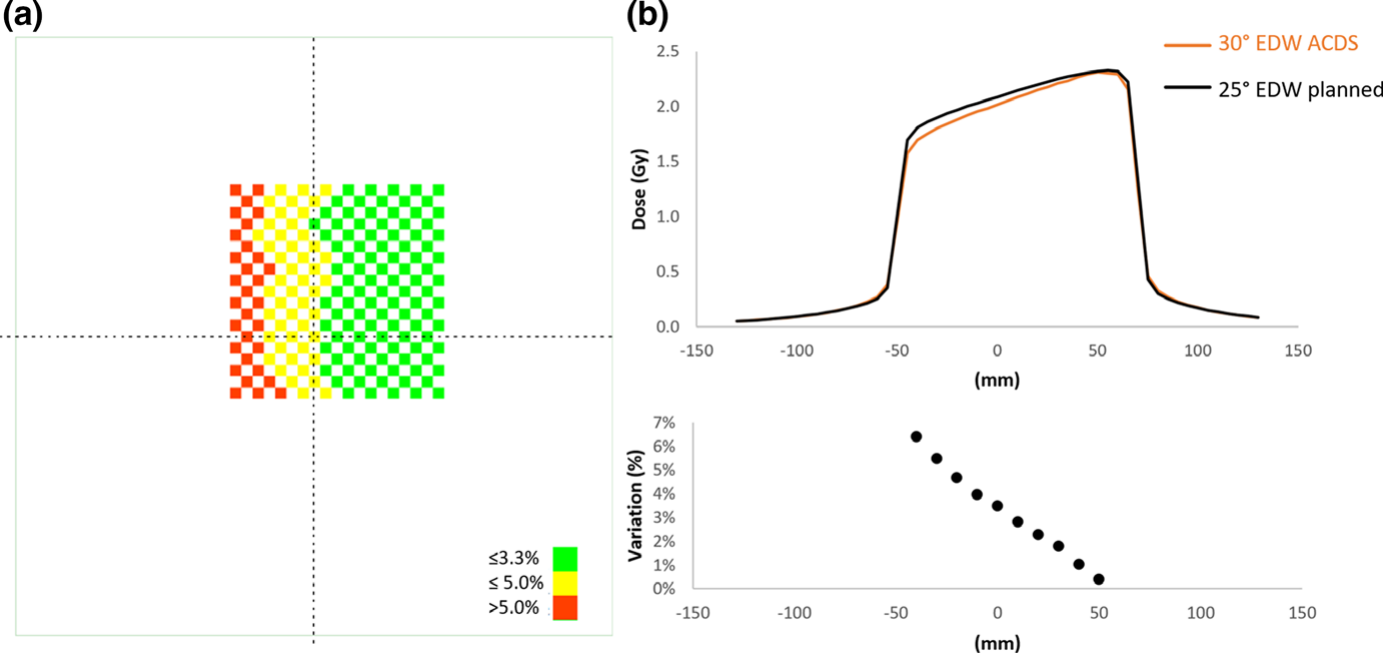
The Australian clinical dosimetry service two‐dimensional results for adjusted wedge plan is shown in panel 3a, and panel 3b shows the left–right dose profile above, and the dose variation across the profile below. The central axis dose difference is 3%, but at the toe of the wedge, the dose discrepancy increases to almost 7%.

In this case of using a 25° EDW instead of a 30° EDW, the ACDS audit show greater sensitivity to the error than IROC's measurement on the central axis. Figure 3 provides insight into the reason behind this. It shows the 2D results from the plan with the introduced error. At central axis, the discrepancy is approximately 3%, but at the toe of the wedge, the discrepancy increases to almost 7%. In this case, the 2D information from the wedge is able to highlight discrepancies from the entire field. The ACDS audit does not correct for the measured daily output. If the ACDS were to correct for the measured daily output of 1.012 cGy/MU for 6X, the results shown in Table 7 would improve, but the results from the plan with introduced errors would still be out of tolerance.

### 3.5 Off‐axis factors

**Figure 4:**
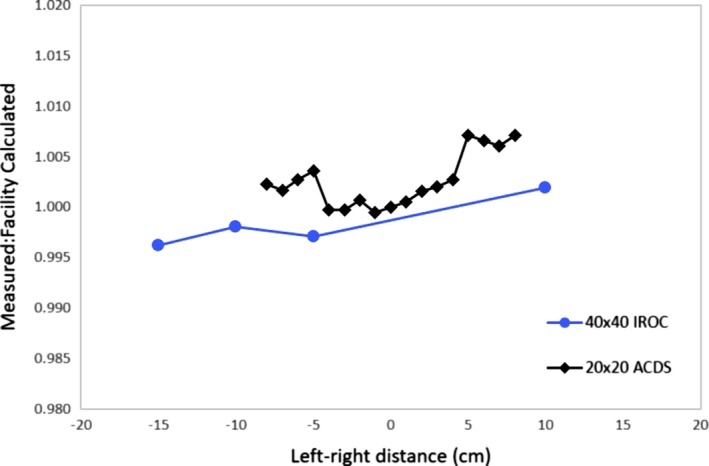
Off‐axis factor variation measured by the Australian clinical dosimetry service and imaging and radiation oncology core in the left–right direction.

Both audits investigate off‐axis factors. As the ACDS audit is array based, every field includes off‐axis factors. However, IROC investigates in a much larger field than ACDS, 40 × 40 cm^2^, compared to the maximum 20 × 20 cm^2^ used by the ACDS. In Fig. 4, the off‐axis factors measured by each group in maximum field size in the left–right direction are compared. Very similar results were seen with both groups, showing very good agreement with the facility plans, and a very small trend with increasing discrepancy from left to right.

### 3.6 Overall outcome

With the original TPS calculated plans, based on the clinical beam model, almost all results were optimal, and almost all findings were consistent between the two audit methodologies. Only two results were different between the results of IROC and the ACDS based on the clinical data. The first was the clinical reference calibration for the FFF beams in the ACDS audit, in which the ACDS categorized as pass (action) whereas IROC categorized as optimal. The measurements of FFF photons showed a slight discrepancy (1.6%) with the institution, and a slight discrepancy with IROC of 0.7% due to the inclusion of a 0.5% nonuniformity correction by the ACDS. As per TG‐51 addendum, the beam nonuniformity should be corrected for with FFF beams if the chamber does not have a short collecting volume.^24^ The second difference between IROC and the ACDS was seen with the lung phantom. The asymmetric field behind lung with and without wedge had a maximum variation of 3.5% and 3.6%, respectively. IROC did not detect this issue because their measurements were all assessed in a homogeneous phantom.

The results can similarly be summarized for the beam model with errors introduced. Neither audit was sensitive enough to pick up a 2% change to the small field output factors. The PDD change was flagged by both audits, as was the introduced error of using 25° wedge instead of 30° wedge.

## 4 Conclusions

Despite different equipment and scope of measurements in on‐site audits, there was notable consistency between the ACDS and IROC audit findings in the reference and basic dosimetry conditions covered in this study. Both audits investigate field size output factors, small field output factors, photons PDD, wedge factors, and off‐axis factors.

The two audit approaches also displayed a similar response when errors were introduced into the treatment planning system. They both flagged the incorrect PDD and wedge data, and neither was able to pick up the 2% change to small field output factors.

There were differences in the methodologies that resulted in detectable but small differences in results. For example, the 2D array used by the ADCS was more sensitive to the particular error introduced in the wedge data as it also measured the greater discrepancy off axis in the toe of the wedge than the corresponding measurements by IROC. The range of PDD measurements performed by IROC was more sensitive to detecting errors in the PDD than the suite of measurements conducted by the ADCS. There were also some audit aspects that were unique, for example, use of a lung slab phantom by the ACDS or the assessment of depth dependence of electron beams by IROC. Overall, these test differences and differences in sensitivities provide an opportunity for growth for each of IROC and the ACDS.

The tests performed in this comparison are only a subset of each group's onsite dosimetry review visits. IROC also conducts a thorough review of the institution's QA procedures and documentation; treatment records, assessment of IGRT capability, and MLC calibration. The scope of the ACDS on‐site visits are focused on dosimetry measurements, but are not limited to fundamental beam modeling and conformal delivery. The ACDS also measures IMRT, VMAT, and SBRT treatment plans, MLC calibration, and IGRT capability while on‐site. Despite the expansion of the audit into advanced modalities, there are continued recommendations generated from the conformal audit component. This fundamental part of dosimetry testing remains relevant to identify flaws in individual implementation of beam models and limitations in commercial algorithms.^29^ The IROC recommendations similarly continue to show fundamental issues, particularly with small field output factors and wedge factors.^9^ The broader scope of IROC on‐site visit also includes QA program review, with shared aspects with the QUATRO clinical audit approach.^30^ Although resource intensive, the broader consideration of radiotherapy practice identified many areas for improvement with substantial numbers of recommendations relating to deficiencies in the QA program.

The similarities between the findings from the two groups across the core dosimetry assessment are encouraging in the context of seeing similar standards and expectations in radiotherapy dosimetry around the world. As we increasingly move to gathering clinical trial data from international trial participants, it is important that there is a harmonized approach to radiotherapy quality assurance and dosimetry audit.
